# Aloe Vera Gel-derived Eye Drops for Alkaline Corneal Injury in a Rabbit Model

**DOI:** 10.18502/jovr.v15i1.5932

**Published:** 2020-02-02

**Authors:** Mohsen Rezaei Moghadam, Mohammad-Reza Jafarinasab, Zahra Yousefi, Ali Sanjari Moghaddam, Hajar Memarzadeh, Mozhgan Rezaei Kanavi

**Affiliations:** ^1^ Ophthalmic Research Center, Shahid Beheshti University of Medical Sciences, Tehran, Iran; ^2^ Torfeh Medical Center, Shahid Beheshti University of Medical Sciences, Tehran, Iran; ^3^ School of Traditional Medicine, Shahid Sadoughi University of Medical Sciences, Ardakan, Iran; ^4^ Ocular Tissue Engineering Research Center, Shahid Beheshti University of Medical Sciences, Tehran, Iran

**Keywords:** Alkali Burn, Aloe Vera, Epithelialization, Rabbits

## Abstract

**Purpose:**

To investigate the efficacy of topical *Aloe Vera* (AV) gel-derived eye drops on the healing of alkali-burned corneas in rabbits.

**Methods:**

Thirty alkali-burned corneas of 30 New Zealand albino rabbits were categorized into three groups: AV treatment group that received AV gel-derived eye drops four times a day; medical therapy (MT) group that received conventional treatment; and the control group. Clinical examinations together with digital imaging of the corneas were performed on days 0, 1, 2, 4, and 7. The area of the corneal epithelial defect (CED) was measured using ImageJ software. After euthanizing the rabbits, the affected corneas were evaluated by histopathological examination. Finally, the clinical and histopathological results were compared among the groups.

**Results:**

The CED area on days 2 and 7 was significantly less in the AV group than that in the MT group (*P* = 0.007 and *P* = 0.024, respectively) and the control group (*P* = 0.003 and *P* = 0.037, respectively). None of the cases developed hypersensitivity reactions, limbal ischemia, descemetocele, or corneal perforation during the study period. Based on histopathology, the AV group had notably less keratocyte loss than the MT group (*P* = 0.001) and the control group (*P* = 0.022). The inflammatory response after the alkali burn was higher in the AV group than that in the controls (*P* = 0.028).

**Conclusion:**

Short-term topical AV treatment was effective in healing alkali-burned corneas and hastened corneal re-epithelialization as compared to MT; however, AV gel-derived eye drops did not reduce the inflammatory response.

##  INTRODUCTION

Ocular chemical burns, with an estimated annual incidence of 50 per 100,000 people, are common and serious ocular injuries that need emergency care.^[1,2]^ The mechanism of injury in ocular chemical burns is production of reactive oxygen species and lack of balance between the inflammatory and anti-inflammatory responses.^[1,3,4]^ Therefore, finding a new ideal material with antioxidant properties may be promising for the treatment of such injuries.^[3]^ *Aloe barbadensis Miller*, commonly referred to as *Aloe Vera* (AV), is a succulent plant that has high water content that ranges from 99 to 99.5%. The remaining 0.5–1.0% of solid material contains over 75 different potentially active compounds including water- and fat-soluble vitamins, minerals, enzymes, simple and complex polysaccharides, phenolic compounds, and organic acids.^[4]^ The biological and medical properties of AV correspond to the synergic effects of different substances, not to a particular one.^[4,5,6]^

Currently, AV has been implemented in the treatment of several disorders such as arthritis, cutaneous burns, cancers, eczema, psoriasis, gastrointestinal disorders, hypertension, diabetes, human immune deficiency virus infections, and genital herpes.^[4]^ There is limited information about the role of AV in corneal epithelial healing; a recent study reported AV as an efficient material that enhances corneal re-epithelialization and reduces the inflammatory response after alkali burns in diabetic rats.^[7]^ Nevertheless, some studies showed no improvement in corneal wounds when treated with AV.^[8]^

Considering the mechanism of injury in ocular chemical burns and the short- and long-term side effects of corticosteroids, which are one of the main conventional treatments, the application of antioxidant agents such as AV may be cost-effective in these injuries, reduce the adverse effects, and improve the anatomical and visual outcomes. This study investigated whether the use of AV gel-derived eye drops was more advantageous than the conventional medical treatment for healing the alkali-burned corneas in a rabbit model.

##  METHODS

##  Animal Models and Randomization 

Thirty New Zealand albino rabbits that weighed approximately 2.5 kg were enrolled in the study under the ARVO Statement for the Use of Animals in Ophthalmic and Vision Research and the Guidelines for Animal Research at the Ophthalmic Research Center, Shahid Beheshti University of Medical Sciences, Tehran, Iran. Before starting the experiment, the animals were examined for the presence of any ocular or systemic disorder. All study procedures were approved by the Ethics Committee of the Ophthalmic Research Center at the Shahid Beheshti University of Medical Sciences (Tehran, Iran). Chemical burns were induced in the right eye of the rabbits under general anesthesia with an intramuscular injection of 10% ketamine HCl (30 mg/kg) (Alfamine; Alfasan, Woerden, Holland) and 2% xylazine (3 mg/kg) (Rompun; Bayer, Leverkusen, Germany). Topical anesthesia was achieved using tetracaine 0.5% eye drops (Anestocaine, Sina Darou Laboratories, Tehran, Iran). A lid speculum was inserted and a 6 mm in diameter round filter paper impregnated with 2.5 N of sodium hydroxide was applied in the center of the right cornea for 30 sec followed by a 3-min irrigation with normal saline solution (0.9% NaCl). Then, the rabbits were randomized into three groups of 10 animals: (a) AV-treated group that received AV gel-derived eye drops four times a day for seven days; (b) medical treatment (MT) group that received a seven-day conventional topical therapy^[9,10,11]^ consisting of chloramphenicol 0.5% eye drops (Chlobiotic, Sina Darou Laboratories) four times a day, betamethasone 0.1% eye drops (Betasonite, Sina Darou Laboratories) four times a day, sodium citrate 10% eye drops (Aurocitrate; Aurolab, Madurai, India) four times a day, ascorbic acid 10% solution prepared from a Vitamin C ampule containing 500 mg/5 ml ascorbic acid (Vitamin C, Daru Pakhsh Pharmaceutical Company, Tehran, Iran) four times a day, and atropine 1% eye drops (Atrin, Sina Darou Laboratories, Iran) once a day; and (c) control group that received sterile normal saline eye drops four times a day for seven days.

##  Preparation of AV Gel-derived Eye Drops

AV gel was removed from the latex of the AV plant in accordance with the guidelines and recommendations from the International Aloe Science Council (http://www.iasc.org). Briefly, identical leaves from a one and a half-year-old AV plant were removed and thoroughly washed. Then, under sterile conditions, 5 cm from the ends of the leaves were removed and the rest of the leaves were divided into 10 cm pieces. After the segregation of the rind, the mesophyll, located between the rind and the central gel parenchyma, was scraped and preserved in a sterile container. Using a needleless 10 mL syringe, the gel was aspirated and pushed out through a 23 mm needle to filter the gel into an ultrasonic homogenizer device (BANDELIN, Berlin, Germany). The homogenized solution was then filtered through a nylon membrane microbiologic filter (pore size, 0.22 µm) into a sterile eye drop bottle. These preservative-free eye drops were kept at 4°C during the study period.

##  Clinical Examinations

All rabbits were examined on days 0, 1, 2, 4, and 7 using an ophthalmic operating microscope (OMS-300; Topcon, Tokyo, Japan). During each examination, the development of any possible complications such as corneal perforation, infection, and allergic reaction was investigated. After fluorescein was instilled, an iPhone 6S equipped with an 8-megapixel digital iSight camera was used to capture images from the corneas and the adjacent metric ruler. ImageJ software (ImageJ 1.48, National Institute of Mental Health; http://rsb.info.nih.gov/ij/) was used to analyze the captured images and calculate the total area of the corneal epithelial defects (CEDs).

##  Histological Evaluation

The rabbits were euthanized on day 7 with an intravenous injection of sodium phenobarbital.^[12]^ The right eyes of all rabbits were enucleated and fixed in 10% neutral buffered formalin for 24 h. The excised corneoscleral tissue was bisected through the affected zone, processed, and embedded into paraffin blocks. Thin tissue sections from five consecutive tissue planes (200 µm apart) were stained with hematoxylin and eosin, periodic acid–Schiff (PAS), and Gram-Twort and examined under light microscopy (BX41, Olympus, Japan) to evaluate epithelial integrity, corneal thinning, stromal neovascularization, keratocyte loss, endothelial cell loss, stromal inflammation, and retrocorneal membrane formation. A digital camera (DP12 Microscope Camera, Olympus, Japan) mounted on the light microscope was used to take images from the central and paracentral zones of the corneas at magnifications 100x and 40x. ImageJ software was used to count keratocytes (magnification 100x) and endothelial cells (magnification 40x) in each tissue plane.^[11,13]^ Photomicrographs were taken under 100 and 40 microscope power-fields, respectively, from the central and paracentral zones of the corneas by using a digital camera (DP12 Microscope Camera, Olympus, Japan) assembled on the light microscope. Keratocytes and endothelial cells were enumerated. The severity of stromal inflammation and loss of keratocytes and endothelial cells were graded according to the histological grading presented in Table 1. Moreover, stromal vascularization was considered significant when central and/or para-central zones of the cornea were vascularized.

**Table 1:** Histological grading for the loss of keratocytes, endothelial cells, and stromal inflammation


**Grading/Histologic Finding**	**Mild**	**Moderate**	**Severe**
**Keratocytes loss**	Mean of 7–10 keratocytes per 100 power-field	Mean of 3–6 keratocytes per 100 power-field	Mean of 0–2 keratocytes per 100 power-field
**Endothelial cell loss**	Mean of 9–12 endothelial cells per 40 power-field	Mean of 3–8 endothelial cells per 40 power-field	Mean of 0–2 endothelial cells per 40 power-field
**Stromal inflammation**	Anterior stromal involvement	Anterior and middle stromal involvement	Full thickness stromal involvement

**Table 2:** Comparison of the mean area of the corneal epithelial defect (CED) among the Aloe Vera (AV), medical therapy (MT), and control groups at the baseline and on days 2, 4, and 7, as well as the changes of CED areas over the seven-day period


**Time**	**Group**			**P${}^{d}$**	**Multiple Comparison**
	**Control (1)**	**MT (2)**	**AV (3)**	
**Baseline (Day0)**	33.25 ± 6.84	30.43 ± 1.05	29.73 ± 1.26	0.265	
**Day1** ${}^{a}$	17.05 ± 10.83	12.04 ± 3.62	10.431 ± 3.79	0.302	
Change 0–1${}^{a}$	16.20 ± 6.16	18.38 ± 3.34	19.30 ± 3.92	0.432	
% of change 0–1${}^{b}$	55.48 (21.33 to 79.65)	59.43 (47.85 to 82.25)	69.71 (45.18 to 78.98)	0.348	
Within P${}^{c}$	0.005	0.005	0.005	
**Day2** ${}^{a}$	8.56 ± 10.05	3.99 ± 2.52	1.31 ± 2.29	0.004	1,3 (0.003)–2,3 (0.007)
Change 0–2${}^{a}$	24.69 ± 4.94	26.43 ± 2.11	28.42 ± 2.91	0.072	2,3 (0.028)
% of change 0–2${}^{b}$	79.20 (33.37 to 97.96)	87.44 (73.68 to 99.16)	98.41 (73.21 to 99.84)	0.006	1,3 (0.004)–2,3 (0.009)
Within P${}^{c}$	0.005	0.005	0.005	
**Day4** ${}^{a}$	3.11 ± 1.54	7.67 ± 3.70	4.30 ± 2.47	0.010	1,2 (0.003)–2,3 (0.049)
Change 0–4${}^{a}$	30.14 ± 7.26	22.75 ± 3.89	25.43 ± 2.70	0.011	1,2 (0.005)–1,3 (0.049)
% of change 0–4${}^{b}$	91.91 (81.22 to 95.73)	74.92 (52.71 to 93.18)	85.13 (74.12 to 99.76)	0.008	1,2 (0.002)–2,3 (0.049)
Within P${}^{c}$	0.005	0.005	0.005	
**Day7** ${}^{a}$	1.78 ± 2.85	1.74 ± 1.91	0.26 ± 0.49	0.043	1,3 (0.037)–2,3 (0.024)
Change 0–7${}^{a}$	31.47 ± 4.86	28.68 ± 1.87	29.47 ± 1.49	0.369	
% of change 0–7${}^{b}$	97.87 (82.78 to 100)	96.52 (83.10 to 100)	100 (94.41 to 100)	0.041	1,3 (0.037)–2,3 (0.024)
Within P${}^{c}$	0.005	0.005	0.005	
AV, Aloe Vera Group; MT, Medical Therapy Group				
Multiple comparisons are performed by the Mann–Whitney method. For multiple comparisons, only *P* < 0.05 are presented.
The area of the defect is in squared millimeters.					
${}^{a}$ The results are presented as the mean ± the standard deviation.					
${}^{b}$The results are presented as the median (range).					
${}^{c}$ The data are based on a Wilcoxon signed-rank test.					
${}^{d}$The data are based on a Kruskal–Wallis test.					

**Table 3:** Comparison of the histopathologic results of the corneal tissues among the Aloe Vera (AV), medical therapy (MT), and control groups


**Histopathologic Features**		**Groups**			**P- Value${}^{a}$**	**Multiple Comparison**
	**Control (1)**	**MT (2)**	**AV (3)**	
Epithelial integrity	Yes	3 (30.0%)	3 (30.0%)	2 (20.0%)	0.999	–
	No	7 (70.0%)	7 (70.0%)	8 (80.0%)	
Neovascularization	Non-significant	10 (100.0%)	10 (100.0%)	9 (90.0%)	0.999	–
	Significant	0 (00.0%)	0 (00.0%)	1 (10.0%)	
Keratocyte loss	None to Mild	4 (40.0%)	0 (0.0%)	9 (90.0%)	0.022	(2 vs 3 *P* < 0.001)
			(1 vs 2 *P* = 0.029)
	Moderate to Severe	6 (60.0%)	10 (100.0%)	1 (10.0%)	(1 vs 3 *P* = 0.022)
Endothelial Cell Loss	Mild	0 (0.0%)	0 (0.0%)	0 (0.0%)	0.999	–
	Moderate to Severe	10 (100.0%)	10 (100.0%)	10 (30.0%)	
Inflammation	None to Mild	8 (80.0%)	5 (50.0%)	3 (30.0%)	0.106	(1 vs 3 *P* = 0.028)
	Moderate to Severe	2 (20.0%)	5 (50.0%)	7 (70%)	
AV, Aloe Vera Group; MT, Medical Therapy Group						

##  Statistical Analysis

Data were presented as the frequency, percentage, mean ± standard deviation, and lower and upper limits. The results within each group were assessed using the Wilcoxon signed-rank test. Kruskal–Wallis and Fisher exact tests were used to compare variables among the groups. To make a pairwise comparison between the groups at a time, the Mann–Whitney U test was implemented. All analyses were performed using SPSS statistical software version 24 (IBM Corp., Armonk, NY). *P* < 0.05 were considered statistically significant.

##  RESULTS

##  Clinical Results

**Figure 1:**
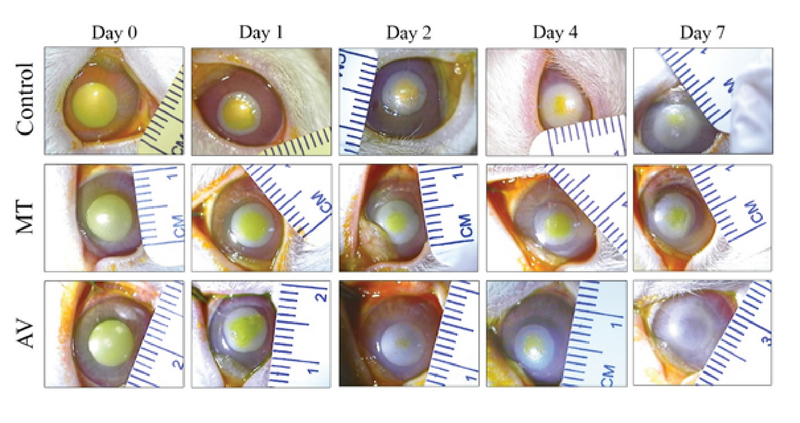
Mean CED areas and changes in the defective areas in the AV, MT, and control groups over the seven-day period are illustrated in the representative graph, showing a greater rate of corneal re-epithelialization in the AV group than that in the other two groups. AV, Aloe Vera; CED, corneal epithelial defect; MT, medical therapy.

**Figure 2:**
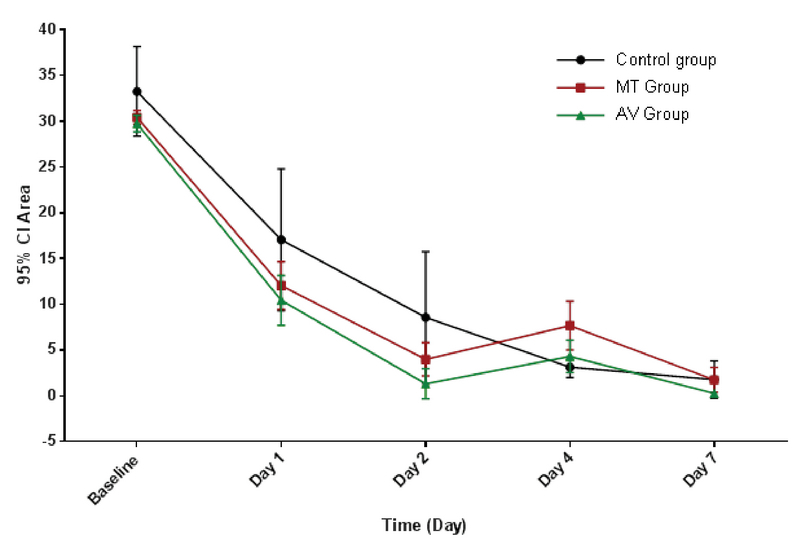
Photographs of fluorescein-stained rabbit corneas following alkaline burn in the AV, MT, and control groups at baseline (day 0) and over the seven-day period.

All 30 rabbits survived during the study. None of the animals showed evidence of allergic reaction, infection, perforation, descemetocele, or limbal ischemia. Table 2 and Figure 1 illustrate the mean area of the CEDs measured on days 0, 2, 4, and 7 and their changes over the seven-day period. The mean CED area was significantly different among the three groups on days 2, 4, and 7 (*P* = 0.004, *P* = 0.01, and *P* = 0.043, respectively) [Figure 2]. The defective area in the AV group on days 2 and 7 was significantly less than that in the MT group (*P* = 0.007 and *P* = 0.024, respectively) and the controls (*P* = 0.003 and *P* = 0.037, respectively). On day 4, the CED area in the AV group was comparable with that in the controls but significantly less than that in the MT group (*P* = 0.049). Compared to the CED area measured on day 0, the reduction in the CED area on day 2 was greater in the AV group than that in the MT group. Moreover, reduction in the CED area measured on day 4 was significantly greater in the controls as compared to the AV and MT groups (*P* = 0.049 and *P* = 0.005, respectively). The corneal defect area measured on day 7 was significantly reduced in all study groups as compared to that measured on day 0 (*P* = 0.005). The reduction in the CED area on day 7 was not significantly different among the groups (*P* = 0.369).

**Figure 3:**
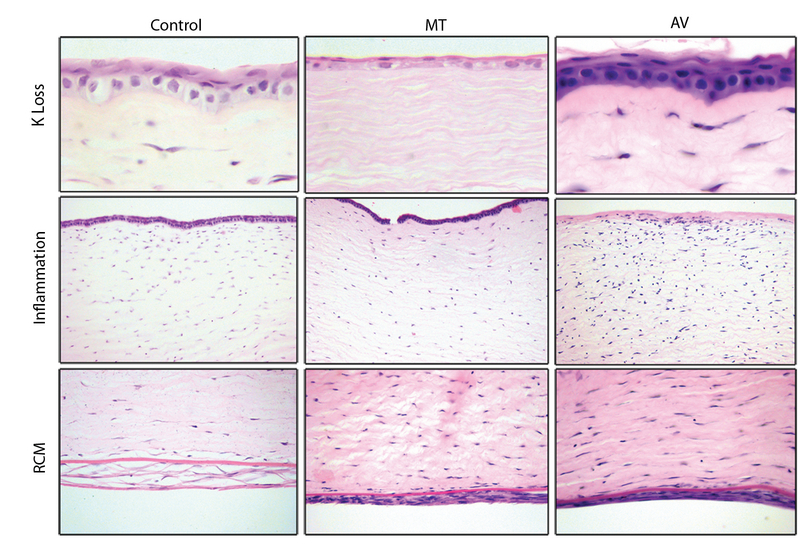
Histopathology of the rabbits' corneas. Note moderate-to-severe keratocyte loss (K Loss) in the MT group compared to the mild K loss in the control and AV corneas (hematoxylin and eosin (H&E) × 1000). Moderate-to-severe stromal inflammation is present in the AV group in comparison to lower degrees of inflammation in the control and MT corneas (H&E × 200). Note the presence of a retrocorneal membrane in the control, MT, and AV corneas, showing a moderate-to-severe endothelial cell loss in all groups (H&E × 400). AV, Aloe Vera; MT, medical therapy

##  Histopathologic Results

Histopathologic examination of the excised corneas [Table 3] showed no significant difference among the groups in terms of epithelial integrity (*P* = 0.999), neovascularization (*P* = 0.999), retrocorneal membrane formation, and endothelial cell loss (*P* = 0.999). None of the cases developed corneal thinning. Retrocorneal fibrous membrane formation along with focal endothelial cell loss were observed in all cases. There was a significant difference among the groups in terms of keratocyte loss (*P* = 0.022), with the AV group demonstrating a lower rate of moderate to severe keratocyte loss compared to the MT (*P* < 0.001) and control (*P* = 0.022) groups. The rate of moderate to severe keratocyte loss was higher in the MT group than in the controls (*P* = 0.029) [Figure 3].

In regard to stromal inflammation, the rate of moderate to severe inflammation was significantly higher in the AV group [Figure 3] than that in the controls (*P* = 0.028). PAS and Gram-Twort-stained slides revealed no microorganisms in any samples.

##  DISCUSSION

Our study demonstrated that AV gel-derived eye drops for alkali-burned cornea were effective in reducing the defective area compared to the conventional treatment and controls. Furthermore, the histopathological results showed that treatment with AV gel-derived eye drops was superior compared to the conventional medical therapy (MT) and controls in terms of a lower rate of keratocyte loss. Similar results were observed in another study,^[7]^ in which AV eye drops, prepared from the AV-lyophilized powder, were effective in rapid re-epithelialization of chemically burned corneas in diabetic rats. Although they reported a decreased rate of inflammation at the microstructural level, the loss of keratocytes was not investigated in their study.

While the rate of re-epithelialization with AV gel treatment was greater than that with MT at all examination time points, the trend of epithelial healing in both AV and MT groups demonstrated an increase in the CED area on day 4. This feature was not observed in the controls, although the mean of CED area on day 4 was not significantly different between the AV gel-treated and control groups. The trend in re-epithelialization observed in our study was similar to that reported by the majority of studies. These studies demonstrated rapid CED healing from days 0 to 2, followed by an epithelial breakdown phase, ^[1,14]^ indicating a regression and enlargement of the CED on day 4.^[14,15]^ This observation is attributable to the lack of formation of desmosomes and microvilli and the presence of differentiation defects in the basement membrane^[14,16]^ that are induced by ocular chemical burns, regardless of the type of treatment.

In this study, the lowest rate of keratocyte loss was observed in the AV group, whereas the MT group had the highest rate of keratocyte loss. This finding is in line with the results of previous studies.^[11,13]^ This is probably due to the antioxidant effect of AV, which protects stromal keratocytes from apoptosis. The preservation of keratocytes maintains corneal tissue stability and prevents complications such as melting and chronic ulcers. Given the implications of AV gel-derived eye drops at the microstructural level observed in this study, it can be concluded that AV was able to promote the preservation of corneal stromal cells, unlike conventional medical treatment and other proposed treatment modalities such as electromagnetic therapy.^[11,13]^

Our results showed non-significant degrees of neovascularization in all groups which was a part of the reparative process secondary to a chemical burn.^[3]^ The histopathological evaluation did not show a significant difference in the rate of stromal neovascularization among the groups. Chemical burns stimulate inflammatory and immune-mediated responses that can lead to angiogenesis.^[17]^ Angiogenesis acts as a double-edged sword in corneal tissue restoration; it can cause long-term complications including corneal edema, lipid deposition, stromal hemorrhage, and scarring that ultimately decrease visual acuity.^[18,19]^ Anti-angiogenic medications such as anti-VEGF have adverse effects on epithelial healing and increase the risk of corneal melting.^[18]^

An anti-inflammatory effect of AV has been documented by Atiba et al^[7]^ in their three-day investigation on corneal alkali burns in diabetic rats. However, in this seven-day study, unlike our expectation, a moderate-to-severe inflammation was observed in the majority (70%) of the AV group compared to the 30% of eyes in the controls. We did not find any microorganisms on PAS and Gram stains, which would have been potentially associated with increased stromal inflammation. We suppose that our study, in comparison with the study by Atiba et al,^[7]^ was long enough to elucidate the significant presence of stromal inflammatory cells in the AV group, which is expected to occur in the acute phase of a reparative process. However, the short nature of our study did not allow us to observe the eradication of inflammation after one week. On the other hand, factors such as the use of a particular part of the AV leaf should not be neglected. In this study, we used the mucilage mesophyll layer of the plant under the rind, which contained the highest concentration of mono- and polysaccharides and potent antimicrobial and analgesic phenolic compounds, such as anthraquinones.^[20,21,22]^ The presence of mono- and polysaccharides in the part of the AV leaves that we used might have been correlated with the occurrence of higher degrees of stromal inflammation in the AV-treated eyes as compared to the other groups.

There are some limitations in this study. The short follow-up time was a major shortcoming that might have confounded the interpretation of our results, particularly the histopathologic outcomes. The lack of apoptotic assays and molecular investigations were another limitation of the study, which warrant further evaluations. Additionally, we used a homogenized and filtered solution of AV gel in our study being unaware of the exact amounts of the active components. Therefore, further investigations should be planned to verify the amounts of these components in the preparation used in the current study.

In conclusion, this study demonstrated the superiority of AV gel-derived preservative-free eye drops compared to conventional therapy for the treatment of corneal chemical burns. AV as a single-drug, low-cost therapy yielded better outcomes, including faster re-epithelialization and a decreased rate of keratocyte loss, compared to multiple drug regimens in the MT group. The observation of moderate-to-severe stromal inflammation with short-term AV treatment might have been either a part of a rapid re-epithelialization process or due to the use of the AV leaf mesophyll. This can be verified in further investigations by using a pharmaceutical-grade AV leaf mesophyll that contains optimized concentrations of polysaccharides.
